# Establishment of the Patient Acceptable Symptom State (PASS) for the Achilles Tendon Total Rupture Score in a Swedish Population

**DOI:** 10.1177/23259671241253280

**Published:** 2024-07-25

**Authors:** Elin Larsson, Sara Brandt Knutsson, Annelie Brorsson, Christer Johansson, Katarina Nilsson Helander

**Affiliations:** † Department of Orthopaedics, Sahlgrenska University Hospital, Institute of Clinical Sciences at Sahlgrenska Academy, Gothenburg University, Gothenburg, Sweden; ‡ IFK Kliniken Rehab, Gothenburg, Sweden; § Department of Orthopaedics, Institute of Clinical Sciences at Sahlgrenska Academy, Gothenburg University, Sweden; Investigation performed at Sahlgrenska University Hospital, Mölndal, Sweden

**Keywords:** Patient Acceptable Symptom State, PASS, Achilles tendon Total Rupture Score, ATRS

## Abstract

**Background:**

As the use of patient-reported outcome measures (PROMs) is increasing in orthopaedic research, there is also a growing need for a standardized interpretation of these scores, such as the Patient Acceptable Symptom State (PASS), defined as the value beyond which patients consider themselves well. The Achilles tendon Total Rupture Score (ATRS) is the only PROM specific for Achilles tendon ruptures.

**Purpose:**

To establish the PASS for the ATRS in a Swedish population.

**Study Design:**

Cross-sectional study; Level of evidence, 3.

**Methods:**

Patients treated for an acute Achilles tendon rupture at a single institution in Sweden (injured between July 1, 2018, and December 31, 2020) were asked to participate in this study. The patients completed a questionnaire consisting of the ATRS and an anchor question: “How satisfied are you with the result of your treatment?” Receiver operating characteristic curve analysis was performed to calculate the PASS threshold for a positive response to the anchor question.

**Results:**

Of 516 eligible patients, 316 (61%) were included. The time from injury to completion of the questionnaire ranged from 12 to 27 months. The PASS threshold for the ATRS was found to be 75. The median ATRS of all patients was 80; 66% of patients reached an ATRS ≥75. Overall, 79% of patients were satisfied with the results of their treatment.

**Conclusion:**

The estimated PASS for the ATRS was 75 in the general Swedish population at 12 to 27 months after an acute Achilles tendon rupture.

The use of patient-reported outcome measures (PROMs) in orthopaedic research has been increasing in recent years. PROMs are self-reported measures designed to collect information about the factors that are important to patients. In joint arthroplasty registers, the standard primary outcome has been revision surgery, which affects only a minority of patients. Therefore, it makes more sense to also measure outcomes that are relevant to all patients, such as pain relief and functional improvement.^37^ The same argument can be applied to Achilles tendon ruptures, for which rerupture has long been the primary outcome of choice. However, the incidence of rerupture is low. In their meta-analysis from 2019, Ochen et al^23^ reported the rerupture rates for surgical and nonsurgical treatment as 2.3% and 3.9%, respectively. Nevertheless, irrespective of treatment, 20% of the patients do not return to their preinjury activity level.^39^ PROMs provide researchers with the possibility to add patients’ experience of disease and injury as well as their treatment to the scientific evaluation.

PROMs can be classified into generic or disease specific. Generic PROMs, such as the EuroQoL-5 Dimension Questionnaire, are focused on general health or health-related quality of life. Disease-specific PROMs are adapted to a particular condition or treatment.^37^ The Achilles tendon Total Rupture Score (ATRS) is the only PROM specific for Achilles tendon rupture.^22^ It was developed in 5 steps: item generation and test construction, item reduction, evaluation of the final ATRS, test-retest, and responsiveness. During the development, the final version was tested for internal consistency, structure, and validity.^22^ It was developed in 2007 and has since then been translated into 14 languages.^‖^

The growing interest for patient-reported results has not come without challenges. How can one know if a statistically significant difference is relevant to patients? Concepts of minimal clinically important difference (MCID) or minimal clinically important improvement (MCII) and Patient Acceptable Symptom State (PASS) have been developed to answer these questions.

The MCID, the smallest change in measurement that patients perceive as beneficial, was first defined by Jaeschke et al^13^ in 1989. The MCII is defined as the smallest change that signifies an improvement.^33^ The MCID can be defined by distribution- or anchor-based methods. Distribution-based approaches are based on statistical criteria from the PROM scores. Anchor-based approaches use an external indicator (anchor), which can be either an objective or subjective measure.^27^

The concept of PASS was originally established by Tubach et al^34^ in 2005 and is defined as the value beyond which patients consider themselves well. The PASS is usually defined by patients answering an anchor question, which is sometimes supplemented by a validating question. The combination of the anchor question and the PROM gives a level above which the patients can be considered satisfied with the result of the treatment.

In 2020, Dams et al^9^ published a study evaluating the Dutch version of the ATRS, including the minimal important change (MIC) between 3 and 6 months after an acute Achilles tendon rupture. The MIC is related to the MCID but is usually defined as the change in health status over time in a single group or single individual.^17^ The MCID was originally developed in rheumatology. The baseline for patients with rheumatologic diseases is easy to define and collect before the start of intervention. For injuries such as an Achilles tendon rupture, the baseline is harder to define. In PROMs, patients are often asked to state their preinjury status retrospectively, but to use this as a baseline for MCID could be questionable. The other alternative is to use a postinjury status as baseline and then compare that status with a later one. In the study by Dams et al,^9^ the 3-month results were used as baseline and compared with the results at 6 months. The PASS, as stated, is the level above which patients consider themselves well. When evaluating injuries and not diseases, to establish the PASS is of greater use.

There is 1 previous study on the PASS for the ATRS, published by Cramer et al^7^ in 2022. The authors evaluated the PASS 6 months, 1 year, and 2 years after an acute Achilles tendon rupture based on data from the Danish Achilles tendon Database. The ATRS PASS was found to be 49 at 6 months, 57 at 1 year, and 52 at 2 years postinjury.^7^

The aim of the current study was to establish the ATRS PASS for patients with an acute Achilles tendon rupture in a Swedish population, which can be useful for clinicians in interpreting the score.

## Methods

The research protocol received Swedish Ethical Review Authority approval, and all participants gave written consent after having been provided written information about the study. Participants were derived from a larger retrospective cohort study of patients being treated for an acute Achilles tendon rupture at a single institution between January 1, 2015, and December 31, 2020. Patients were identified by the registration of the International Classification of Diseases code for an acute Achilles tendon rupture (S86.0) or a spontaneous flexor tendon rupture (M66.3). The patients’ hospital records were controlled to verify the diagnosis. Patients were treated either surgically or nonsurgically. The choice of treatment was decided by the physician in consultation with the patient according to local guidelines.

All patients in the cohort who were injured between July 1, 2018, and December 31, 2020, (30 months) were contacted by mail and invited to participate in the study. The questionnaires were sent out to patients on 2 separate occasions, October 2020 and December 2021, meaning that the time from injury to completion of the questionnaire ranged from 12 to 27 months.

The patients were asked to answer a questionnaire consisting of ATRS and an anchor question. The questionnaire was completed either physically on paper or electronically. Patients who had not answered the questionnaire within 2 weeks received a new mail with a reminder to complete the questionnaire. Patients who still had not completed the questionnaire 1 week after the second mail then received a reminder by telephone. This was to ensure that the patients had received the information.

### Outcome Measures

#### Swedish Version of the ATRS

The ATRS consists of 10 questions about patients’ symptoms and limitations after their Achilles tendon injury. Each question is answered on an 11-point (range, 0-10) scale, which gives a total maximum score of 100. A score of 100 indicates no symptoms and full recovery.

#### Anchor Question

The anchor question was a modified version of the question used by Tubach et al.^35^ Patients were asked (translated from Swedish to English), “How satisfied are you with the result of your treatment?” Answers were graded on a 5-point scale: 1 = completely satisfied, 2 = satisfied, 3 = neither satisfied nor dissatisfied, 4 = dissatisfied, or 5 = completely dissatisfied. Completely satisfied and satisfied were considered positive responses, while all other responses were considered negative.

### Statistical Analysis

Statistical analysis was performed using Stata statistical software (Release 17; StataCorp). Data were reported as means and standard deviations for age and body mass index and as medians with interquartile ranges (IQRs) for ATRS values. Categorical variables were reported as frequencies and percentages.

Receiver operating characteristic (ROC) curve analysis was performed to identify the threshold for the ATRS that predicted a positive response to the anchor question. The area under the curve (AUC) of a test is equal to the probability that the test will rank a randomly chosen positive outcome higher than a randomly chosen negative one. The AUC ranges from 0.5 (equivalent to a test with no accuracy in predicting the outcome variable) to 1.0 (a perfectly accurate test in identifying the outcome variable in the total population).^10^ An AUC >0.7 is considered acceptable discriminating power, while an AUC >0.8 is considered excellent.^29^

The PASS cutoff score was determined according to the Youden index.^30^ The threshold was the ATRS where sensitivity and specificity are maximal in predicting the outcome variable.

## Results

There were 516 patients eligible for inclusion in the study, of whom 316 (61%) were included, having answered both ATRS and the anchor question. The mean age of the participants was 51 years (range, 18-92 years), and 73 (23%) were women. In total, 99 (31%) patients were treated surgically. The characteristics of the study cohort are presented in Table 1.

**Table 1:** Descriptive Characteristics of the Study Population (N = 316)^*a*^

Characteristic	Value
Age, y	51 ± 15.4
Sex	
Female	23 (73)
Male	77 (243)
Body mass index, kg/m^2^	26.2 ± 3.8
Type of treatment	
Nonsurgery	69 (217)
Surgery	31 (99)

a Data are reported as mean ± SD or % (n).

**Figure 1.**
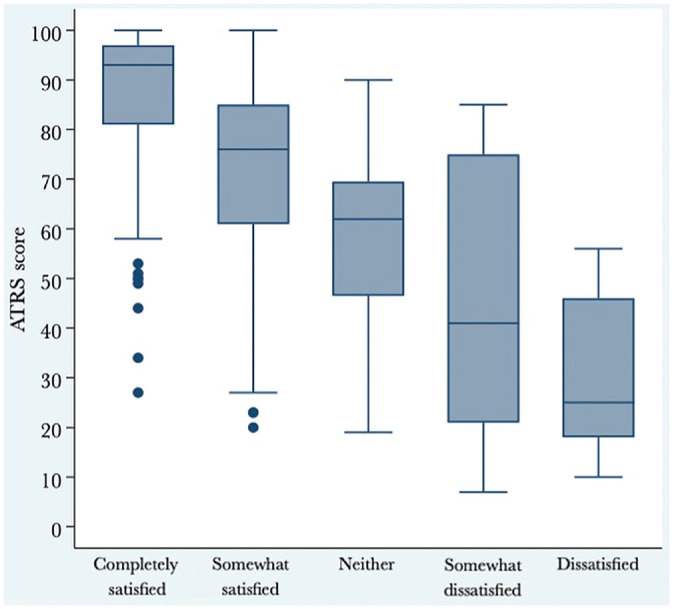
Box plot displaying ATRS distributions at 12 to 27 months after injury according to anchor question response categories. The box indicates the interquartile range (the middle 50% of scores) and the line in the box indicates the median. The dots outside the minimum whisker indicate outliers (ie, >1.5 times the IQR). One-way analysis of variance was used to determine whether there were significant differences in ATRS values among the 5 response groups. Bonferroni correction was applied in the post hoc analysis. The post hoc analysis identified a statistically significant difference between all groups (*P* < .001). ATRS, Achilles tendon Total Rupture Score; IQR, interquartile range.

**Figure 2.**
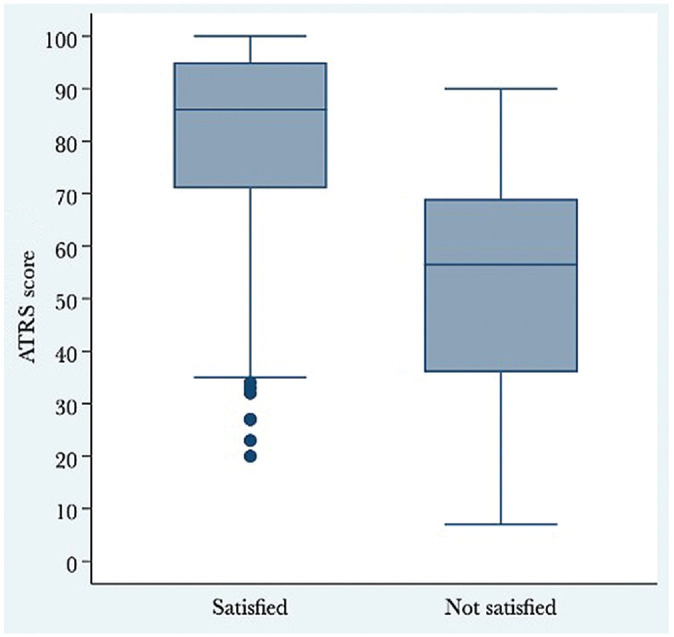
Box plot displaying ATRS distributions at 12 to 27 months postinjury after dichotomizing the original anchor question response categories. The box indicates the IQR (the middle 50% of scores) and the line in the box indicates the median. The dots outside the minimum whisker indicate outliers (ie, >1.5 times the IQR). The difference between the groups was statistically significant (*P* < .001, Mann-Whitney *U* test). ATRS, Achilles tendon Total Rupture Score; IQR, interquartile range.

The median ATRS of all patients was 80. As shown in Table 2, 79% of patients were satisfied with the result of their treatment. Figure 1 illustrates the ATRS distributions at 12 to 27 months postinjury in terms of anchor question response categories. Among the patients satisfied with their treatment, the median ATRS was 86 (IQR, 71-95). Among the patients not satisfied with their treatment, the median ATRS was 57 (IQR, 36-69) (Figure 2).

**Table 2:** ATRS Values According to Anchor Question Responses at 12 to 27 Months After Injury^*a*^

Anchor Question Response	Patients, % (n)	ATRS, Median (IQR)
Response		
1 (completely satisfied)	45 (143)	93 (81-97)
2 (somewhat satisfied)	34 (106)	76 (61-85)
3 (neither satisfied nor dissatisfied)	14 (44)	62 (47-70)
4 (somewhat dissatisfied)	5 (16)	41 (21-75)
5 (dissatisfied)	2 (7)	25 (18-46)
Response categories		
Satisfied (1-2)	79 (249)	86 (71-95)
Not satisfied (3-5)	21 (67)	57 (36-69)

a ATRS, Achilles tendon Total Rupture Score.

**Figure 3.**
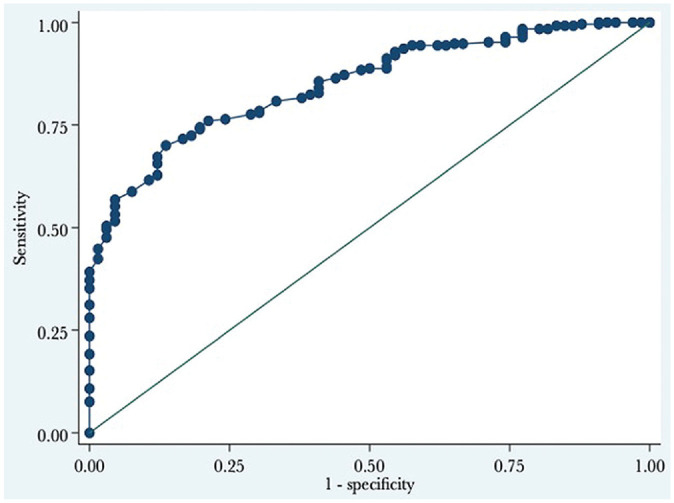
The receiver operating characteristic curve for the PASS for the ATRS at 12 to 27 months postinjury. The PASS value is where the sum of the sensitivity and specificity is maximized, which is the Youden index. ATRS, Achilles tendon Total Rupture Score; PASS, Patient Acceptable Symptom State.

As presented in Figure 3 and Table 3, the ATRS PASS threshold was 75. Overall, 66% of patients reached an ATRS ≥75, and 72% of patients who were considered satisfied with their treatment reached the PASS. On the other hand, 18% of the patients who were considered not satisfied reached the PASS. The AUC was 0.85 and thus considered excellent (Table 3).^29^

**Table 3:** AUC and PASS for the ATRS^*a*^

	AUC (95% CI)	Sensitivity	Specificity	PASS Cutoff^*b*^	*P*
ATRS	0.85 (0.80-0.89)	0.70	0.86	75	<.001

a ATRS, Achilles tendon Total Rupture Score; AUC, area under the curve; PASS, Patient Acceptable Symptom State.

b Determined according to the Youden index.

## Discussion

The most important finding of this study was that the ATRS PASS threshold value was 75 at 1 to 2 years after an acute Achilles tendon rupture in a Swedish population; 66% of the study patients reached this level. The ATRS PASS value in this study was higher than the value proposed by Cramer et al^7^ in a Danish population, which was 57 at 1 year and 52 at 2 years after injury. In this study, we used the ROC curve method of calculating the PASS, whereas Cramer et al used the predictive modeling method. It has been suggested that the latter is more precise. However, to our knowledge, ROC curve analysis is still the most common method. Cramer et al published an appendix with calculations of PASS with the ROC curve method. The threshold values were up to 4 points lower with the ROC curve method. Thus, when using the same method, the difference between the studies was even bigger.

This difference in ATRS PASS between the 2 studies raises questions. The mean age and proportion of men and women were equal between the 2 studies. However, while 31% of patients were surgically treated in our study, only 8% (1-year follow-up group) and 14% (2-year follow-up group) had surgery in the study by Cramer et al.^7^ Nevertheless, in the most recent randomized controlled trial (RCT), Myhrvold et al^19^ did not find that surgical treatment was associated with better outcomes regarding patient recovery compared with nonsurgical treatment.

In the current study, we chose to analyze all patients in the same group, regardless of time from injury. The time from injury to completion of the questionnaire ranged from 12 to 27 months. The rationale for this was the previous finding that only minor improvements occur between the 1- and 2-year evaluations in a Swedish population.^25^ However, it should be noted that Cramer et al^7^ did note a decrease in PASS threshold of 5 points between 1 and 2 years in a Danish population. The wording of the anchor question also differed between the 2 studies. We chose to specifically ask about satisfaction with the result of treatment, whereas Cramer et al^7^ asked, “Taking into account all the activities you have during your daily life, your level of pain, and also your functional impairment, do you consider that your current state is satisfactory?” This difference could have affected the results.

There was also a difference in the ATRS values in these 2 studies that is not explained by the different wording of the anchor questions. The median ATRS in our study was 80. Cramer et al^7^ did not state the median ATRS for all patients, but the ATRS values for the satisfied patients were 73 at 1 year and 74 at 2 years postinjury. This is inferior to the median/mean ATRS reported in previous studies.^4,12,16,19,21,23,24^ In the largest published Achilles tendon rupture RCT so far, Myhrvold et al^19^ found a mean ATRS at 12 months of 77 to 79, irrespective of treatment. In their 2019 meta-analysis, Ochen et al^23^ listed the included studies reporting ATRS (5 studies in total): Nilsson-Helander et al^21^ found mean ATRS values at 1 year of 88 and 86 in the surgical and nonsurgical groups of their RCT, respectively. Olsson et al,^24^ in their RCT, reported a mean ATRS of 82 for surgically treated and 80 for nonsurgically treated patients. An observational study by Jackson et al^12^ found that the median ATRS was 94 in the surgical and 82 in the nonsurgical group. In a questionnaire follow-up of 487 patients, Bergkvist et al^4^ reported a mean ATRS of 82 for the surgical and 79 for the nonsurgical group. Lim et al,^16^ in a prospective study of 200 patients, found a mean ATRS of 85 for all patients.

Metz et al^18^ evaluated patients up to 1 year after an acute Achilles tendon rupture in their RCT and found visual analog scale scores for satisfaction of 7.5 for patients treated either surgically or nonsurgically. In a retrospective study of patients treated nonsurgically, Lerch et al^15^ found that 94% of patients stated that they were satisfied with the treatment. When Svedman et al^31^ assessed if reduced time to surgery affects ATRS, they used an ATRS ≥80 as a threshold for an overall good subjective outcome. However, the reasons for using this value as a cutoff were not explained.

### Limitations

Regarding study limitations, these calculations are based on a Swedish population and are therefore not suitable for use in other countries. It is crucial to do separate calculations for the PASS in different countries. In addition, it is important to bear in mind the possible bias of 39% of the eligible patients who did not answer both questions. It is difficult to comment on whether this could contribute to an overestimated or underestimated value of the PASS. However, this was a relatively high response rate. Cramer et al^7^ presented data based on 3 populations with response rates of 51%, 50%, and 57%, respectively.

The concept of PASS is not to be seen as a definitive level of patient satisfaction. The threshold is the ATRS, where sensitivity and specificity are maximal in predicting an outcome variable. Thus, in the studied population, an ATRS of 75 is the level at which both sensitivity and specificity for satisfaction are at their highest. However, the ATRS PASS reflects the population in which the study took place. This affects the generalizability of the results. It is also possible that this is one of the explanations for the difference in ATRS between our study and that of Cramer et al^6^ on ATRS PASS. As previously mentioned, a population of healthy, active nonsmokers will probably have a different PASS than a more inert group. Nevertheless, in a general population comparable to ours, we believe that an ATRS PASS of 75 is plausible.

## Conclusion

The estimated PASS value of ATRS was found to be 75 in the general Swedish population 12 to 27 months after an acute Achilles tendon rupture. The estimated threshold value was higher than in the single previous study on ATRS PASS.^7^ Discussed explanations for this included an overall higher median ATRS as well as different wordings of the anchor question. Our belief is that these new findings regarding ATRS PASS will be useful for clinicians’ interpretation of their patients’ ATRS.

## Footnotes

‖ References 1-3, 5, 6, 8, 11, 14, 20, 26, 28, 32, 36, 38.
